# Quantitative Association of Anatomical and Functional Classes of Olfactory Bulb Neurons

**DOI:** 10.1523/JNEUROSCI.0303-18.2018

**Published:** 2018-08-15

**Authors:** Andrej Tavakoli, Anja Schmaltz, Daniel Schwarz, Troy W. Margrie, Andreas T. Schaefer, Mihaly Kollo

**Affiliations:** ^1^Behavioural Neurophysiology, Max Planck Institute for Medical Research, 69120 Heidelberg, Germany,; ^2^Department of Neuroscience, Physiology and Pharmacology, University College London, WC1E 6BT, London, United Kingdom,; ^3^Behavioural Neurophysiology Laboratory, Francis Crick Institute, NW1 1AT, London, United Kingdom,; ^4^Department of Anatomy and Cell Biology, Faculty of Medicine, University of Heidelberg, 69120 Heidelberg, Germany,; ^5^Division of Neurophysiology, Medical Research Council National Institute for Medical Research, NW7 1AA, London, United Kingdom,; ^6^Sainsbury Wellcome Centre for Neural Circuits and Behaviour, University College London, W1T 4JG, London, United Kingdom, and; ^7^Department of Neuroradiology, Heidelberg University Hospital, Heidelberg, 69120, Germany

**Keywords:** cluster analysis, external tufted cell, interneuron, juxtaglomerular, olfactory bulb, periglomerular

## Abstract

Juxtaglomerular cells (JGCs) of the olfactory bulb (OB) glomerular layer (GL) play a fundamental role in olfactory information processing. Their variability in morphology, physiology, and connectivity suggests distinct functions. The quantitative understanding of population-wise morphological and physiological properties and a comprehensive classification based on quantitative parameters, however, is still lacking, impeding the analysis of microcircuits. Here, we provide multivariate clustering of 95 *in vitro* sampled cells from the GL of the mouse (male or female C57BL/6) OB and perform detailed morphological and physiological characterization for the seven computed JGC types. Using a classifier based on a subselection of parameters, we identified the neuron types in paired recordings to characterize their functional connectivity. We found that 4 of the 7 clusters comply with prevailing concepts of GL cell types, whereas the other 3 represent own distinct entities. We have labeled these entities horizontal superficial tufted cell (hSTC), vertical superficial tufted cell, and microglomerular cell (MGC): The hSTC is a tufted cell with a lateral dendrite that much like mitral cells and tufted cells receives excitatory inputs from the external tufted cell but likewise serves as an excitatory element for glomerular interneurons. The vertical superficial tufted cell, on the other hand, represents a tufted cell type with vertically projecting basal dendrites. We further define the MGC, characterized by a small dendritic tree and plateau action potentials. In addition to olfactory nerve-driven and external tufted cell driven interneurons, these MGCs represent a third functionally distinct type, the hSTC-driven interneurons. The presented correlative analysis helps to bridge the gap between branching patterns and cellular functional properties, permitting the integration of results from *in vivo* recordings, advanced morphological tools, and connectomics.

**SIGNIFICANCE STATEMENT** The variance of neuron properties is a feature across mammalian cerebral circuits, contributing to signal processing and adding computational robustness to the networks. It is particularly noticeable in the glomerular layer of the olfactory bulb, the first site of olfactory information processing. We provide the first unbiased population-wise multivariate analysis to correlate morphological and physiological parameters of juxtaglomerular cells. We identify seven cell types, including four previously described neuron types, and identify further three distinct classes. The presented correlative analysis of morphological and physiological parameters gives an opportunity to predict morphological classes from physiological measurements or the functional properties of neurons from morphology and opens the way to integrate results from *in vivo* recordings, advanced morphological tools, and connectomics.

## Introduction

A fundamental property of mammalian cerebral networks is their broad functional and morphological tuning (Parra et al., 1998; Markram et al., 2004; Marder, 2011; Kepecs and Fishell, 2014). Projection neurons (PNs) and interneurons (INs) exhibit striking morphologic variance, rendering the definition of primary cell types a daunting task. Even within cell sets of relative morphological homogeneity, the physiotype may considerably differ (Schaefer et al., 2003; Helmstaedter et al., 2009a; Druckmann et al., 2013), increasing the diversity of cellular response properties, likely serving computational purposes (Padmanabhan and Urban, 2010; Angelo and Margrie, 2011). Similarly, neurons with common immunohistochemical markers express a range of features in morphology or physiology (Krimer et al., 2005; Kiyokage et al., 2010). These findings have shaped the view that neuronal diversity results from the combination of an abundance of continuous properties, such that cells sharing common attributes may feature great variability in others.

Functional and morphological variability is also present within the olfactory bulb, the first processing stage of olfactory information in the brain, constraining the large-scale study of its neuronal circuits. In the most superficial olfactory bulb (OB) layer, the axons of olfactory sensory neurons (OSNs), and dendrites of PNs and INs form densely packed spherical structures (the olfactory glomeruli) where synaptic transmission from OSN axons to PNs is organized (Mombaerts, 1996; Shepherd et al., 2004; Helmstaedter, 2013). Neurons within the glomerular layer (GLs) are diverse and commonly subsumed under the term juxtaglomerular cell (JGC). Qualitative morphology defined initially three types of JGCs: external tufted, periglomerular (PGC), and superficial short axon cells (Pinching and Powell, 1971). Further investigation has revealed a startling number of subtypes, with studies grouping cells according to classifiers, including morphology, immunohistochemical profile, physiology, connectivity, ion channel expression, and targeted glomerular compartment (Wachowiak and Shipley, 2006; Kosaka and Kosaka, 2007; Panzanelli et al., 2007; Parrish-Aungst et al., 2007; Whitman and Greer, 2007; Shao et al., 2009; Fried et al., 2010; Sawada et al., 2011), such that the original terminology falls short of capturing the actual heterogeneity. For efficient circuitry analysis and study comparability, however, a population-wise accurate description of cell types is crucial (Denk et al., 2012; Seung and Sümbül, 2014). Creating comprehensive, publicly available databases of the various properties of statistically sampled neurons would thus greatly advance the *in vivo* study of neuronal circuits (Mott and Dingledine, 2003).

Here we investigate the cluster-separating power of standard morphological and physiological parameters for neurons of the OB GL and explore the predicting power of physiological parameters on morphological classes. We performed whole-cell patch-clamp recordings from *n* = 95 GL neurons in brain slices and used *post hoc* biocytin staining to reveal their detailed morphology. During data analysis and within Results, we avoid generic terminology to prevent bias toward established cell classes. While multiparametric analysis, such as cluster analysis (CA) of neurons, has been performed routinely in other areas of the brain (Cauli et al., 2000; Chou et al., 2010), its application within the OB was limited to subclasses of neurons (Eyre et al., 2008; Kollo et al., 2014), rather than a global, random sample of all elements of the circuit. We therefore performed CA of multiple physiological and morphological parameters to objectively specify the class “JGC” beyond the terms “external tufted,” “periglomerular,” and “superficial short axon cell.” Next, we used this dataset to train a classifier based on a combination of both *in vitro* and *in vivo* easily attainable physiological and morphological parameters to reliably identify cell class. Finally, we used this model to predict the identities of *n* = 35 neuron pairs with clear dendritic projection to a common target glomerulus to study the synaptic connectivity between neurons in different clusters.

## Materials and Methods

### 

#### 

##### Slice preparation.

All experimental procedures were performed according to the animal welfare guidelines of the Max Planck Society. Male or female C57BL/6 mice (MGI catalog #5656552, RRID:MGI:5656552) (P35–P42) were anesthetized with isoflurane (Baxter Deerfield), decapitated, and the brain surgically removed within ice-cold slicing solution (in mm as follows: 125 NaCl, 25 NaHCO_3_, 25 glucose, 2.5 KCl, 2 MgCl_2_, 1.25 NaH_2_PO_4,_ 1 CaCl_2_, sparged with 95% O_2_/5% CO_2_). The mouse brain was cut horizontally in ice-cold slicing solution at 300 μm thickness using a vibration microtome (Microm HM 650V, Thermo Fisher Scientific). We incubated slices at 37°C in an incubating chamber containing extracellular solution for 30–50 min and kept the slices for recordings at room temperature for a maximum of 4 h.

##### Pipettes and solutions.

For the recordings, we placed the slices in constantly renewed CO_2_ sparged extracellular solution (in mm as follows: 125 NaCl, 25 NaHCO_3_, 25 glucose, 2.5 KCl, 2 CaCl_2_, 1.25 NaH_2_PO_4_, 1 MgCl_2_, sparged with 95% O_2_/5% CO_2_). Pipettes with 1–2 μm opening diameter and 4–10 mΩ resistance depending on cell size were pulled from 2.0-mm-diameter borosilicate glass capillaries (Hilgenberg) using a DMZ universal puller (Zeitz Instruments). We filled pipettes with an intracellular solution containing the following (in mm: 130 KMeSO_4_, 10 HEPES, 7 KCl, 2 ATP-Na, 2 ATP-Mg, 0.5 GTP, 0.05 EGTA, adjusted to pH 7.4 with KOH, osmolarity 293 mOsm). For the visualization of cells during recording, we added 0.8 mm of fluorescent dye Alexa-488 or AlexaFluor-594 hydrazide (Thermo Fisher Scientific) to the intracellular solution, whereas for *post hoc* neuron reconstruction, we added biocytin (Sigma-Aldrich) at a concentration of 2 mg/ml as histological stain, filling the cells during 2–25 min of recording.

##### Electrophysiological measurements.

For imaging neurons during experiments, we used an Examiner D1 differential interference contrast microscope with automated revolving filters (Carl Zeiss). The GL was identified at the surface of the OB slices as round structures in the autofluorescent signal in the red channel with a W-Plan apochromat 40×/1.0 WDIC objective (Carl Zeiss). For single-cell experiments, we targeted a randomly selected visible neuron within the GL or at the transition of the GL to the external plexiform layer (EPL). Whole-cell patch-clamp mode was established at a pipette pressure of 10 mbar with seal resistances >5 GΩ. Access resistance varied with cell size: for the smallest cells (<7 μm diameter) being <20 mΩ; for larger cells <10 mΩ. Electrophysiological measurements were obtained using a MultiClamp 700B microelectrode amplifier (Molecular Devices) and live-monitored with a 5103N oscilloscope (Tektronix). The data were digitized at 33 kHz by an ITC 18 data acquisition interface (InstruTech), low-pass-filtered at 10 kHz, and recorded with custom written software (Neuromatic, RRID:SCR_004186) plugin to the software Igor Pro (Wavemetrics, RRID:SCR_000325). Before and after break-in, fast and slow capacitive transients, as well as series resistance, were compensated. For current-clamp experiments, series resistance was compensated for using the bridge balance function and capacitance compensation.

##### Paired-recording experiments.

To perform recordings of neuron pairs, we loaded each of the two pipettes with a different fluorescent dye (Alexa-594 and Alexa-488 hydrazide) to distinguish between the two dendritic trees. After whole patch-clamp recording of the first neuron was established and a standard protocol was run (1–2 min), fluorescence filters were used to visualize and follow the dendritic tuft of the neuron to its innervating glomerulus. Next, we targeted a second potentially connected neuron in immediate proximity to this glomerulus using a different fluorescence dye. If clear dendritic tree overlap of both neurons in a common glomerulus was present, we elicited action potentials (APs, 200 nA, 3 ms) in the first neuron in current-clamp mode, whereas the second was held in voltage-clamp mode at a holding potential of −50 mV and monitored for synaptic currents. Next, we repeated the procedure with the reversal of the two neurons to investigate putative connections in the opposite direction (Urban and Sakmann, 2002). At the end of recordings, epifluorescent image stacks were acquired with each filter setting, and the 3D image stacks were *post hoc* again thoroughly checked for dendritic overlap.

##### Histological procedures.

As previously described, the biocytin-filled neurons were processed using standard procedures (Feldmeyer et al., 2005). After recording, slices were carefully blotted with filter paper, transferred to PBS containing 4% PFA, and kept at 4°C for at least 48 h and a maximum of 7 d. After fixation, slices were washed in PBS and processed in 0.1% Triton X-100 solution containing avidin-biotinylated HRP (ABC-Elite; Camon). Next, we used DAB as reactive chromogen until cell processes were well visualized (10 min). In some instances, we enhanced the visibility of cells with 0.5% osmium tetroxide (OsO_4_) for 2–5 min before mounting the slices on glass slides and embedding them in Moviol (Clariant). Finally, 3D digital neuron reconstruction was conducted manually using a Neurolucida setup and software (MicroBrightField, RRID:SCR_001775; <100× magnification).

##### Quantitative measurements.

For each of the reconstructed neurons, we measured 9 morphological parameters using the built-in functions of the Neurolucida explorer (MicroBrightField, RRID:SCR_001775). For the complete list of parameters and definitions, see Table 1. The quantification of electrophysiological parameters was conducted with custom-written procedures in the software Igor Pro (Wavemetrics, RRID:SCR_000325), which we subsequently ran on each of the 95 neuron physiologies. The complete list and definitions of physiological parameters can be found in Table 3. Pseudocode for the acquisition of electrophysiological parameters and data sheets with the complete morphological and physiological parameters for all 95 cells are made available online at https://figshare.com/s/7da895122de6ea83e655.

##### Statistical analysis.

If not otherwise noted, we report all values throughout this work as mean ± SD. All statistical procedures were performed using R (version 3.2.3, Wooden Christmas Tree, 2015 R Foundation for Statistical Computing). For morphological assessment, we used cluster analysis on 9 morphological parameters for all 95 neurons. For physiological assessment, we likewise performed cluster analysis on 9 physiological parameters of the same 95 neurons. Before cluster analysis, morphological parameters were inverse hyperbolic sine-transformed, and all parameters were scaled. For linkage of the cells, we used Ward's method with Euclidean distance measure. For a definition of the correct number of clusters, the gap statistic method was used (Tibshirani et al., 2001). To prove the stability of each cluster, a bootstrapping-based clusterwise assessment of cluster stability was performed (Hennig, 2007).

Before pairwise comparisons of all parameters for each of the morphological clusters, we performed the Shapiro–Wilk normality test of residuals. If the test proclaimed a cluster's residuals to be normally distributed, the QQ plot for the parameter was viewed and visually checked for divergence from a normal distribution. Because most parameters turned out to be non-normally distributed, the nonparametric pairwise Wilcoxon rank-sum test was used. As correction method for pairwise comparisons, we applied the false discovery rate method (Benjamini and Yekutieli, 2001). The complete results for the pairwise Wilcoxon rank-sum tests are provided in the online repository at https://figshare.com/s/7da895122de6ea83e655.

For cluster similarity and dissimilarity evaluation (see Fig. 3*C*), a Euclidean distance dissimilarity matrix between the *n* = 95 cells was calculated. Next, distances were averaged according to each cluster configuration, excluding distance measures of zero along the diagonal of the matrix between self-referenced cells. The original nonaveraged full intercell distance matrix for both parameter spaces is provided in the online repository under the above provided link.

Soma size was measured by the area enclosed by the boundaries of the soma traced under the microscope. Diameters of cells in Table 5 were defined as the shorter diameter of an ellipsoid with an aspect ratio of 1.3 and area identical to the measured soma area.

**Figure 1.**
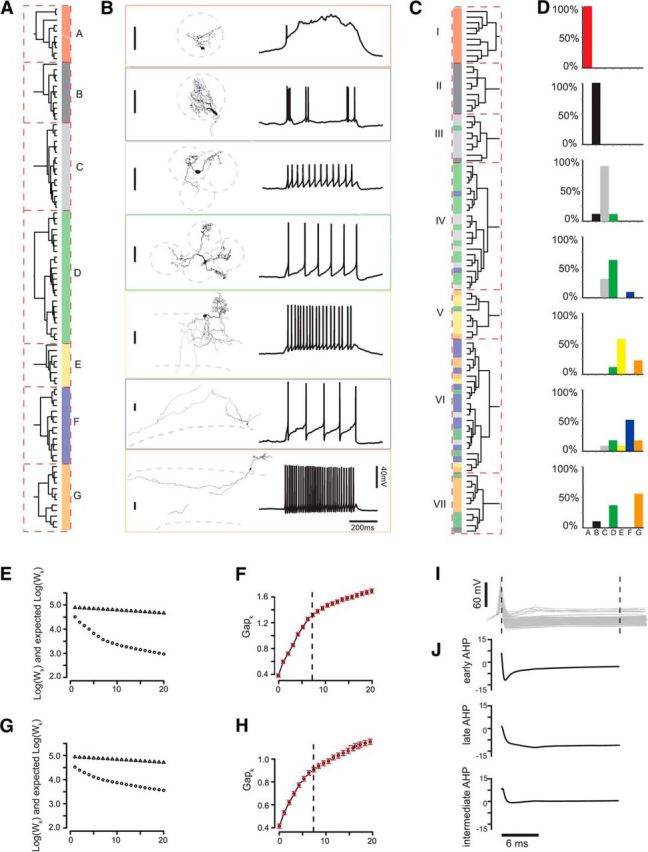
Clustering of juxtaglomerular neurons based on morphological and physiological parameters. ***A***, Cluster analysis (Ward's method, Euclidean distance) of 9 morphological parameters (for parameter distribution, see Fig. 2*A*) yields the depicted dendrogram. Dashed red boxes represent the *n* = 7 significant cell clusters as defined by the gap statistic (Tibshirani et al., 2001). Cluster labels (A–G) and colors are used throughout the study to identify the clusters. ***B***, Example cells from each cluster and its corresponding physiotype (response to a square current pulse). Vertical scale bars represent a distance of 50 μm. ***C***, CA of 9 physiological parameters as shown in Figure 2*B* yields *n* = 7 distinct clusters as defined by the gap statistic. Color bar at each dendrogram leaf represents the origin morphological cluster of each cell. ***D***, Distribution of morphotypes A–G within each physiological Cluster I-VII from ***C***. ***E***, ***F***, Scree plot of the morphological cluster analysis. W_k_ represents the within-sum of squares, Gap_k_ the difference between the Log of W_k_ and expected Log of W_k_ from a reference distribution (Tibshirani et al., 2001). Red bars represent the SE of Gap_k_. The correct number of clusters was chosen at the smallest number of *k* where the double (*f* = 2) SE of *f* added to *f* was larger than the next local maximum of *f*(*k* + 1). ***G***, ***H***, Scree plot for physiological cluster analysis. The correct number of clusters again was found using the *f*.SE rule with *f* = 2. ***I***, Superimposition of all *n* = 95 average spike shapes aligned at spike peak. ***J***, Three resulting factors from the independent component analysis performed on all shown spike shapes. Factor 1 best characterizes the early AHP, Factor 2 the late AHP, and Factor 3 the intermediate AHP.

For the characterization of AP waveforms (see Fig. 1*I*,*J*), we used independent component analysis (ICA) as a method of signal separation on average spike shapes of each neuron (Kollo et al., 2014; Jordan et al., 2018). The code implemented in R was based on the FastICA algorithm described in detail previously (Hyvärinen and Oja, 2000). We used the resulting statistically independent factors to directly represent afterhyperpolarization (AHP) shapes of all neurons as three independent components and labeled these components early, middle, and late AHP throughout this work. To acquire the average spike shapes, the original data traces were scanned for APs during current injection, aligned at the AP maximum, and average spike shapes were calculated. We subsequently aligned all 95 average spike shapes at their maximum. Normalization across average spike shapes regarding the resting membrane potential was achieved by subtracting the voltage at 1 ms before the spike peak from each average spike waveform.

To identify the cell classes of the pair experiments, we used a support vector machine model (SVM, e1071 package, R) based on five physiological parameters and one morphological parameter. These six parameters were chosen to be easily attainable from the pair experiment physiological data. We split the original dataset of 95 cells into training (85%) and test sets (15%), which were subsequently used to train and validate the performance of the SVM model (prediction accuracy = 93.3%, cost = 100, gamma = 0.01). The model was then used to predict the identities of *n* = 52 cells from the pair experiments.

To validate pair connections between two cells projecting dendrites to the same glomerulus, we measured the integral of the postsynaptic current response over a 50 ms time window following the onset of the evoked spike in the presynaptic cell. Next, the current baseline integral was subtracted. For statistical validation of the response, we calculated the current integral of a random 50 ms time window (excluding the 100 ms after the real spike onset) and subtracted the current baseline integral. Last, a paired *t* test was performed on the two data sets (*p* < 0.05); exact *p* values are provided in the online repository at https://figshare.com/s/7da895122de6ea83e655.

## Results

### Objective morphological parameters quantify GL cell anatomy

To date, no multivariate study exists quantitatively examining morphological and physiological JGC features. Because our goal was an objective population-wise classification of GL cell types, we acquired a broad sample of JGCs containing both PNs and INs. We first investigated morphological qualities of all cells by measuring standard morphological parameters that would best differentiate neuron classes (Fig. 1). We defined nine parameters characterizing differences in cellular anatomy (Table 1). For basic representation of the extensiveness of dendritic arbors, the total volume of cell processes and the total dendritic length were chosen. Although inherently correlated, the two parameters diverge in cells with short voluminous dendrites and cells with long or abundantly ramifying thin dendrites. The next class of parameters regarded the extensiveness of neuron ramification: While the convex hull characterizes the total 3D branching of a cell, the furthest Sholl intercept measured the 2D longest distance of any cell process from the soma. Therefore, the two parameters distinguished between concentric and more polarized cells. The sensory input to the GL terminates in a modular pattern in olfactory glomeruli (Mombaerts, 1996). Therefore, the third parameter of this group was defined as the number of contacted glomeruli (Kiyokage et al., 2010), a factor with a strong potential impact on the computational role of a given neuron. The next class of parameters considered the difference in neurons with processes within the EPL. One parameter measured the volume of a cell's potential lateral dendrite located parallel to the GL. In contrast to this, the vertical dendritic volume captured characteristic local dendritic trees in the EPL directly below the cell (Antal et al., 2006). The last two parameters were aimed at measuring more complex cellular details and were created by combining parameters. First, the dendritic density was defined as the quotient of process volume and the convex hull, differentiating between compact and far-reaching dendritic arbors. The second was defined as the product of the neuron somatic size and the process volume as a robust measure of the proximal dimensions of the neuron.

**Table 1.** Morphological parameter definitions

Parameter	Definition
Lateral dendritic volume	Volume of dendrites in the EPL, where a line through the start and endpoint of the dendrite runs parallel (<30°) to the GL-EPL border
Dendritic length	Total dendritic length of the neuron
Soma × dendritic volume	Area of the soma multiplied by the volume of the largest dendrite
Process volume	Volume of all processes of the neuron
Furthest Sholl intercept	Distance from soma to largest sphere intercepted by a dendrite
Vertical dendritic volume	Volume of dendrites in EPL where >85% of dendritic volume is located within a cone with an aperture of 60° and axis perpendicular to the GL-EPL border
Convex hull	Volume of the smallest convex 3D shape fit around a neuron
No. of glomeruli	No. of glomeruli that were passed through by the processes of the cell (Kiyokage et al., 2010)
Dendritic density	Quotient of the biggest GL dendrite of the cell and the convex hull volume
Surface	Volume of the smallest convex 2D shape fit around a neuron

### Cluster analysis of morphological parameters yields seven neuron clusters

**Figure 2.**
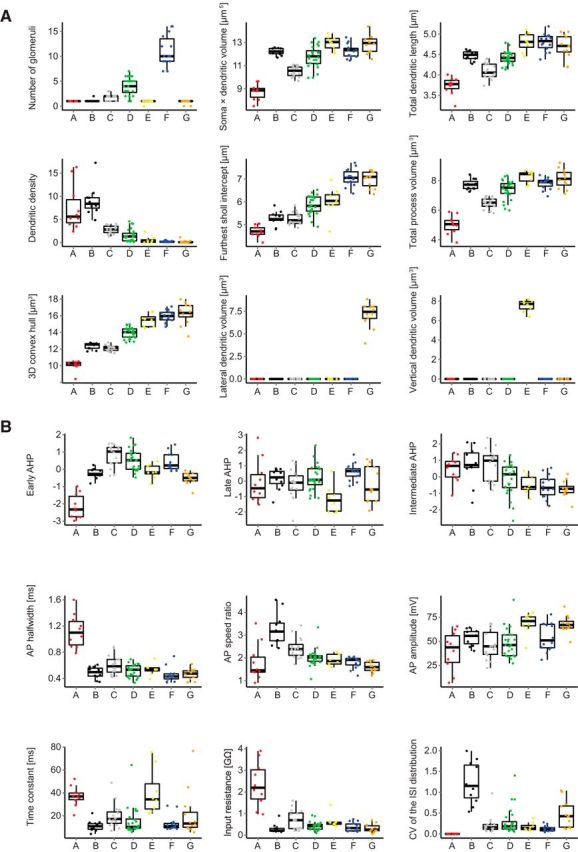
Box plots of physiological and morphological parameter distributions. ***A***, Morphological parameters with color code according to morphological clusters. ***B***, Physiological parameters with color code according to morphological clusters.

In certain neuron populations, cluster analysis of morphological properties has allowed common definitions of distinct neuron clusters, allowing assumptions about the functional roles of these morphologically standardized neurons in their respective circuitry (Cauli et al., 2000; Krimer et al., 2005; Antal et al., 2006; Eyre et al., 2008; Helmstaedter et al., 2009b; Chou et al., 2010). To learn more about the underlying groups within our data, we subjected the nine morphological parameters to hierarchical CA (Ward's method, Euclidean distance), yielding the dendrogram shown in Figure 1*A*. The input parameter distributions are shown in Figure 2*A* and Table 2. To find the correct number of clusters, the gap statistic method was used (Tibshirani et al., 2001) with the *f*.SE rule as a criterion (*f* = 2), which separated *k* = 7 distinct cell clusters within the data, which also corresponded to the inversion point at 7 clusters in the respective Scree plot (Fig. 1*E*,*F*). To assess the likelihood of random, functionally irrelevant clusters within our data, we performed a cluster stability analysis (Hennig, 2007) on the morphological cluster analysis from Figure 1*A*. The resulting Jaccard Index values from this analysis for the seven clusters were calculated as follows after *n* = 500 iterations: A = 0.82; B = 0.82; C = 1.0; D = 1.0; E = 0.9, F = 0.95; and G = 0.93, thus indicating high stability for all clusters (i.e., a high reproducibility of the clusters after many iterations). Thus, it seems highly unlikely that any of the classes A–G have been clustered together by coincidence.

**Table 2.** Morphological parameters of *n* = 95 neurons grouped according to the morphological cluster analysis*^a^*

	A, red (*n* = 10)	B, black (*n* = 11)	C, gray (*n* = 16)	D, green (*n* = 24)	E, yellow (*n* = 8)	F, blue (*n* = 14)	G, orange (*n* = 12)
Process volume (μm^3^)	88 ± 51	1215 ± 475	339 ± 390	955 ± 524	2049 ± 716	1303 ± 440	2041 ± 1315
Convex hull (μm^3^ × 10^3^)	13 ± 5	124 ± 41	95 ± 34	668 ± 418	3318 ± 2527	5278 ± 3568	10053 ± 9679
Lateral dendritic volume (μm^3^)	0 ± 0.0	0 ± 0.0	0 ± 0.0	0 ± 0.0	0 ± 0.0	0 ± 0.0	1062 ± 941
Vertical dendritic volume (μm^3^)	0 ± 0.0	0 ± 0.0	0 ± 0.0	0 ± 0.0	1060 ± 519	0 ± 0.0	0 ± 0.0
No. of glomeruli	1.0 ± 0.0	1.1 ± 0.3	1.8 ± 0.7	3.9 ± 1.7	0.9 ± 0.4	11.0 ± 3.0	0.8 ± 0.4
Soma × dendritic volume (μm^5^ × 10^3^)	3.8 ± 2.3	103.7 ± 28.6	19.9 ± 8.2	93.9 ± 0.7	242.8 ± 133.5	128.5 ± 83.1	253.6 ± 223.2
Dendritic length (μm)	469 ± 168	1973 ± 442	966 ± 377	1821 ± 6	4057 ± 1721	3977 ± 1679	3403 ± 1779
Dendritic density (× 10^−3^)	7.49 ± 4.89	8.99 ± 3.19	2.81 ± 0.86	1.51 ± 1.35	0.55 ± 0.80	0.23 ± 0.16	0.20 ± 0.33
Sholl intercept (μm)	57 ± 14	103 ± 31	101 ± 29	189 ± 72	243 ± 139	624 ± 235	575 ± 184

*^a^*For morphological parameter definitions, see Table 1. A–G indicate clusters as defined by morphological cluster analysis in Figure 1*A*.

After cluster analysis, the seven groups were *post hoc* labeled as morphological Clusters A–G, and this nomenclature was retained throughout this study (Fig. 1*A*). The top four clusters (A = 10, B = 11, C = 16, D = 24) joined after a linkage distance of 16 (data not shown). The bottom three clusters (E = 8, F = 24, G = 12) also joined after an linkage distance of 16, indicating that Clusters A–D shared morphological similarities, as did Clusters E–G. Clusters A–D consisted of small- to medium-sized local cells (Fig. 1*A*,*B*), all of which had their cell bodies and dendrites within the GL. Cluster A (red) contained the altogether smallest cells of the dataset with a small, densely branching dendrite confined to a small volume within one glomerulus. Cluster D (green) cells represented the other extreme of the four clusters, with several dendrites ramifying sparsely over longer distances and contacting more glomeruli (3.9 ± 1.7) than any of the other three top clusters. Cluster C (gray) was comprised of intermediate-sized cells that contacted 1 to 3 glomeruli, although their process volume was the second smallest neurons of the four clusters. Cluster B (black) was represented by cells that abundantly ramified within one glomerulus, consistent with their confined average convex hull volume while process volume was largest among the top four clusters.

The bottom three Clusters E–G (Fig. 1*A*,*B*) included large extensive cells with somata within the GL and on the GL/EPL border and with processes within the GL, the EPL, and the internal plexiform layer. Cluster E cells (yellow) were best characterized by a dense, frequently branching tuft projecting to just one glomerulus and dense local dendrites within the EPL directly below the cell body. Cluster G cells (orange), on the contrary, were defined by their lateral dendrite within the EPL but parallel to the GL. These lateral dendrites branched less frequently than the vertical dendrites of Cluster E cells, which was also consistent with the length of the longest EPL segment being more than twice as large for Cluster G cells as for Cluster E cells. Cluster F cells (blue) were confined to the GL, bore neither lateral nor vertical dendrites, and represented the most extensively ramifying cells of the GL. An example cell of each cell cluster accompanied by an example physiology trace is displayed in Figure 1*B*.

### Electrophysiological properties of the Clusters A–G

There have been numerous attempts to correlate morphological and physiological attributes of cells. While some morphological attributes or classes were found to correlate with distinct cell physiologies, notable intraclass variability was reported (Dumitriu et al., 2007; Helmstaedter et al., 2009a; Chou et al., 2010; Druckmann et al., 2013). In other studies, particularly regarding INs, a clear correlation between neuronal morphology and physiology was not found, leading to the notion that INs express a continuum of properties (Parra et al., 1998; Maccaferri and Lacaille, 2003; Sethupathy et al., 2013; Nagel and Wilson, 2016). After the unsupervised definition of the seven morphological cell clusters within our data, we next investigated how the physiological behavior of the same neurons would relate to their respective morphological identity. We used standard stepwise current injection protocols to elicit APs in all cells and subsequently measured six active and passive standard physiological parameters from the data (Tables 3, 4; Fig. 2*B*). To create three further parameters that represent the AP AHP in detail (Fig. 1*I*,*J*), ICA was performed on AP voltage averages over a time window of 25 ms (Kollo et al., 2014). Previous studies reported that external tufted cell (ETC) bursts undergo a rundown effect after minutes of recording (Hayar et al., 2004b, 2005). We aimed to achieve robust ETC identification by using individual spike properties only to avoid bias from vanishing bursts.

**Table 3.** Physiological parameter definitions

Parameter	Definition
Input resistance (GΩ)	Quotient of the voltage difference to the baseline during stepwise current injection divided by the respective current amplitude
AP half-width (ms)	The difference between the time t_halfmax_ anteceding the spike peak and t_halfmax_ succeeding the spike peak
AP amplitude (mV)	The difference between AP maximum and AP threshold
Time constant (ms)	The time where the voltage response to a step current injection decays by 1/e
CV of the ISI distribution	CV of the interspike interval distribution
AP speed ratio	Quotient of spike rise velocity and the absolute value of spike decay velocity
Spike rise velocity (mV/ms)	The first derivative of the voltage at t_halfmax_ preceding the spike peak
Spike decay velocity (mV/ms)	The first derivative of the voltage at t_halfmax_ succeeding the spike peak

**Table 4.** Physiological parameters of *n* = 95 neurons grouped according to the morphological cluster analysis*^a^*

	A, red (*n* = 10)	B, black (*n* = 11)	C, gray (*n* = 16)	D, green (*n* = 24)	E, yellow (*n* = 8)	F, blue (*n* = 14)	G, orange (*n* = 12)
Early AHP	−2.16 ± 0.71	−0.23 ± 0.29	0.78 ± 0.63	0.52 ± 0.60	−0.12 ± 0.41	0.44 ± 0.50	−0.51 ± 0.35
Late AHP	−0.04 ± 1.38	0.15 ± 0.70	−0.20 ± 0.97	0.22 ± 0.85	−1.17 ± 0.92	0.61 ± 0.53	−0.21 ± 1.19
Intermediate AHP	0.47 ± 0.79	0.73 ± 1.12	0.70 ± 0.98	−0.14 ± 0.98	−0.47 ± 0.57	−0.58 ± 0.69	−0.73 ± 0.51
AP half-width (ms)	1.12 ± 0.25	0.49 ± 0.09	0.60 ± 0.15	0.51 ± 0.10	0.54 ± 0.09	0.45 ± 0.11	0.47 ± 0.08
Time constant (ms)	37.1 ± 8.7	11.1 ± 4.8	19.7 ± 10.5	15.3 ± 12.2	40.7 ± 20.1	13.4 ± 6.7	21.4 ± 20.5
Input resistance (GΩ)	2.34 ± 1.02	0.3 ± 0.21	0.71 ± 0.45	0.42 ± 0.20	0.65 ± 0.31	0.37 ± 0.20	0.33 ± 0.20
AP speed ratio	1.80 ± 0.81	3.24 ± 0.71	2.43 ± 0.45	2.02 ± 0.38	1.92 ± 0.25	1.81 ± 0.25	1.60 ± 0.23
CV of the ISI distribution	0 ± 0.0	1.22 ± 0.49	0.21 ± 0.22	0.30 ± 0.32	0.17 ± 0.10	0.13 ± 0.07	0.47 ± 0.33
AP amplitude (mV)	40.0 ± 20.4	54.1 ± 8.2	46.3 ± 14.7	48.1 ± 14.9	68.3 ± 11.6	54.4 ± 14.5	67.7 ± 8.9

*^a^*For morphological parameter definitions, see Table 3. A–G indicate clusters as defined by morphological cluster analysis in Figure 1*A*. ISI, Interspike interval.

Cluster A cells exhibited a characteristic plateau potential following a fast AP (Fig. 1*B*, red), whereas quantitative parameters were also distinct (Table 4): The input resistance for Cluster A cells was more than threefold larger than for all other morphological cell clusters (for all *p* < 0.001, pairwise Wilcoxon rank-sum test corrected for multiple testing, unpaired), whereas the AP half-width was twice as large as the value of the second highest group, Cluster C (for all *p* < 0.001), also significantly larger than for all other clusters. The majority of Cluster B cells showed bursting spikes with fast, high-frequency APs, which rode on lasting depolarizations (Fig. 1*B*, black). The most characteristic parameter for Cluster B was the AP speed ratio, which on average was larger than the AP speed ratio of all other clusters (for all *p* < 0.05), indicating a fast AP rise and a slow AP decay. The second parameter significantly differing for Cluster B from all other cells was the CV of the interspike interval distribution, representing the irregular occurrence of the spikes (for all *p* < 0.005). Cluster C cells fired evenly distributed APs (Fig. 1*B*, gray) and were also well defined by their average AP speed ratio, which was second highest in the dataset and differed from all other clusters (*p* < 0.05). Input resistance was the second highest on average, and only differed from Cluster A. The locally extensive Cluster D cells were best characterized by the early AHP shape, which differed from all cells (all *p* < 0.05), except Cluster G. Average input resistance and AP speed ratio were not characteristic and did not differ from other cells, except Cluster A and G, respectively.

Cluster E cells had the largest average value for the membrane time constant differing from Clusters B, C, D, and F (all *p* < 0.05). They had the third highest average input resistance of the clusters and had the highest average AP amplitude of all clusters (68.32 ± 11.6 mV, not significant). Cluster F cells were characterized by evenly distributed spikes with the lowest average CV of interspike interval distribution. The AP speed ratio (1.81 ± 0.25) was at the lower end of the range (1.07–4.55), while the input resistance was the third lowest compared with the other clusters. Cluster G cells had the second highest CV of interspike interval distribution, differing from Cluster A, B, and F (all *p* < 0.005), indicating a more irregular spiking pattern than all other clusters. Cluster G cells also had the second highest AP amplitude, which was higher than all other clusters, except Cluster E and F (*p* < 0.05 for all, except E and F).

### Physiological clusters correlate with the defined morphological groups A–G

To assess whether functional properties also characterize well-separated neuron groups, we performed cluster analysis of the physiological parameters. The resulting dendrogram is depicted in Figure 1*C* and yielded *k* = 7 significant clusters (*f*.SE = 2; Fig. 1*G*,*H*) for the same 95 neurons. Figure 1*D* depicts the composition of the physiological Clusters I–VII with the color code matching the morphological Clusters A–G. Together, 67.4% of the cells were grouped with the same neighboring cells they were linked with in the original morphological CA (Fig. 1*A*,*C*,*D*). Physiological Cluster I consisted of cells from Cluster A only, and similarly Cluster II contained cells from Cluster B only. Cluster III consisted of mostly Cluster C cells (7 of 9, 78%). The majority of Cluster IV cells originated from Cluster D (61%, 14 of 23), whereas 30% (7 of 23) of the neurons had the morphological identity of Cluster C (30%). Cluster V comprised mainly (67%, 6 of 9) of neurons from Cluster E. The largest of the clusters, VI, included mostly cells from Cluster F (50%, 12 of 24) but also contained heterogeneous cells from Cluster C (8%), D (17%), E (8%), and G (17%). The majority of the cells in Cluster VII stemmed from Cluster G (55%, 6 of 11), whereas there was a minority of Cluster D cells of 36% within the cluster. As a measure of the likeness of the morphological and physiological clustering, we calculated the Rand Index adjusted for chance, which showed a correlation of the morphological and the physiological clusters of *r* = 0.37. To further examine the exact contribution of each cluster to this global correlation, sensitivity, specificity, and accuracy for the neurons of Clusters A–G were calculated: Cluster A [sensitivity = 100% (10 of 10), specificity = 100% (85 of 85), accuracy = 100% (95 of 95)]; Cluster B [100% (9 of 9), 98% (84 of 86), 98% (93 of 95)]; Cluster C [78% (7 of 9), 90% (77 of 86), 88% (84 of 95)]; Cluster D [61% (14 of 23), 88% (63 of 72), 81% (77 of 95)]; Cluster E [67% (6 of 9), 98% (84 of 86), 95% (90 of 95)]; Cluster F [50% (12 of 24), 97% (69 of 71), 85% (81 of 95)]; and Cluster G [55% (6 of 11), 93% (78 of 84), 88% (84 of 95)].

**Figure 3.**
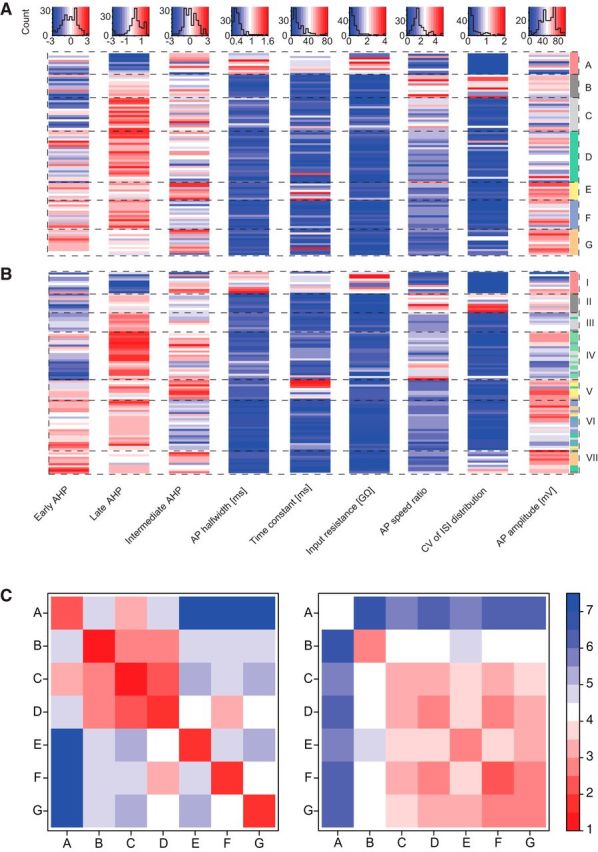
Quantitative association of morphological and physiological parameters grouped according to the morphological clusters (A–G). ***A***, Physiological parameters (columns) of all cells (rows) grouped according to morphological clusters. Top, Histograms represent the distribution of cell count according to each physiological parameter. Color code from dark red to dark blue within the histograms represents the respective parameter values. The row order of the heatmap matches the leaf order of the dendrogram in Figure 1*A*. ***B***, Same as in ***A***, with the row order according to the physiological cluster analysis from Figure 1*C*. ***C***, Clusterwise averages of Euclidean distance measures between all pairings of the *n* = 95 cells (except self-pairings) in the morphological (left) and the physiological parameter space (right).

A complete summary of physiological parameter values for all cells grouped according to the morphological and the physiological clusters is provided in Figure 3*A* and Figure 3*B*, respectively. To assess the similarity of properties within and between clusters, the mean Euclidean distances of cells in a given morphological cluster from the cells of each cluster were calculated in either the morphological or the physiological parameter space (Fig. 3*C*). Morphological parameters displayed high similarity within each morphological cluster and a clear distinction between different clusters (Fig. 3*C*, left). The extent of separation between classes with respect to physiological parameters was more variable (Fig. 3*C*, right). Cluster A and B cells were well separated from other clusters because of their unique functional properties, thus allowing reliable morphological cluster predictions from physiology. On the contrary, Clusters C, D, and G displayed overlapping physiological features and similarity in the mean distance from cells in other clusters, deeming direct inference from solely physiological data less feasible. The remaining Clusters E and F had intermediate physiological similarity measures compared with the other clusters. These results indicate that for the reliable identification of all cell morphotypes, a method combining some morphological information with physiological data, is necessary.

### Machine learning allows prediction of cell cluster identity

**Figure 4.**
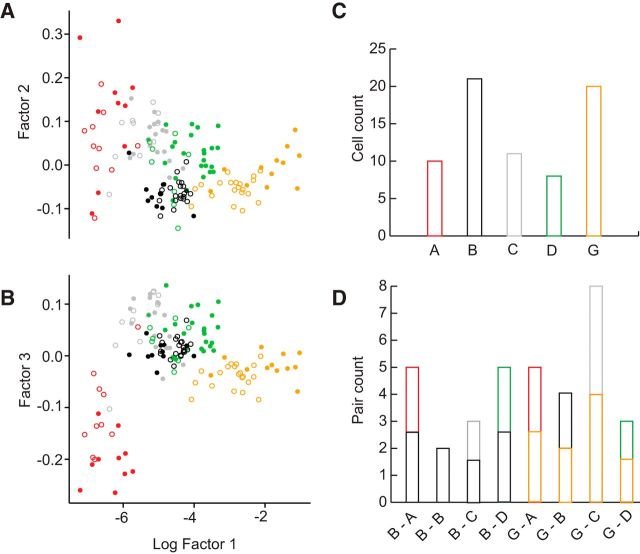
Predictions of cell pair identities of an SVM model based on parameters obtained during recording. ***A***, ***B***, Scatter plot of the three most prominent factors of a singular value decomposition of the original data (filled circles, *n* = 95) and the predictions by the model (empty circles, *n* = 70). An SVM model of the original data was trained as classifier for the predictions with the input parameters early AHP, input resistance, AP speed ratio, AP width, AP amplitude, and 2D convex hull from epifluorescent or reconstructed images. Response vector labels were identical to the results of the morphological cluster analysis. Colors of the empty circles represent the cell class as predicted by the model. Cells from Clusters E and F have been included in the training of the SVM model but were omitted from this scatter plot for clarity. ***C***, Histogram distribution of the *n* = 70 pair experiment cells according to the predicted class. ***D***, Histogram distribution of the *n* = 35 cell pair configurations as result of the predictions. All pairs projected their dendritic tree to a common target glomerulus.

In complex electrophysiological experiments, the recovery rate of cell morphologies can often be low. Knowledge of morphological cell classes, indicative of synaptic connectivity, however, may be pivotal for the interpretation of functional cellular properties. Our data suggested a strong predictive relationship for some of the cell clusters between morphology and physiology and vice versa (Clusters A and B), whereas others could not be identified reliably by physiology alone because the extent of separation between physiology and morphology was less clear (Clusters C, D, and G; Figs. 1, 3). To achieve higher prediction accuracies, which would suffice to predict the morphological class even for these physiologically more variable clusters, we added a single morphological parameter to the set, which was measurable even if a *post hoc* reconstruction of a cell was not successful. Thus, we next assessed whether cell identities could be reliably predicted from a combination of morphological and physiological parameters, which could be easily collected during complex experiments. As a means of objective cell classification, an SVM model was used (Fig. 4). The model was based on the original dataset of 95 cells with one morphological and five physiological parameters. The physiological dimensionality was reduced due to limited time for single-cell data acquisition during complex experiments. Physiological input parameters were early AHP, AP speed ratio, input resistance, AP amplitude, and AP width. Morphology was represented by the 2D convex hull of the cell acquired from epifluorescence image stacks, and the response vector classes were equal to the results of the morphological cluster analysis. After random data separation into a training and test set (85% and 15%, respectively), the model was trained using C-classification and a radial SVM-Kernel (cost = 100, gamma = 0.01). The resulting model prediction accuracy of the test set was 93.3% correctly identified cells.

### Objective *post hoc* definition of cluster pair configurations allows standardized evaluation of pair experiments

To gain further insight into the functional properties of the clusters, we next asked which cell types were connected via direct excitatory synapses. We obtained simultaneous recordings from *n* = 35 neuron pairs with clear dendritic projection to a common target glomerulus as identified visually *post hoc* on epifluorescent image stacks, elicited APs in one of the neurons with high-amplitude current injections while monitoring the current response in the second, voltage-clamped cell. Subsequently, functional connectivity was assessed in the reverse direction (Urban and Sakmann, 2002). The total number of potential interconnections for 7 clusters is 28. Therefore, the chance of recording from a pair consisting of two cells from two defined clusters at any given experiment would be merely 3.57%. To reliably identify the cell type in paired-recording experiments, the SVM model based on the single-cell experiments as described above (Fig. 4) was used. Of the 70 pair experiment cells, neurons were classified by the model to belong to the following classes: A = 10; B = 21; C = 11; D = 8; E = 0; F = 0; G = 20 (Fig. 4*A–C*; these only include cells where successful pair experiments, i.e., both cells projecting to the same glomerulus, could be established; see below). Detailed results of the predictions on the pair cells within the context of the original data are displayed as scatter plots of three factors of a singular value decomposition in Figure 4*A*, *B*. These 70 cells presented themselves in 8 different cluster pair configurations within the 35 paired recordings (B/A = 5; B/G = 4; B/C = 3; B/D = 5; B/B = 2; G/A = 5; G/C = 8; G/D = 3; Fig. 4*D*). The model-based approach allowed a standardized evaluation of the pair experiments with regard to the objectively defined cluster combinations.

Even though all cells were sampled for the evaluation of the pair connections, no pair containing a Cluster E or F cell was investigated. These cells were not excluded a priori. However, no experiments could be established where such cells projected to the same glomerulus as another recorded neuron. There are two likely sources of this bias: First, the somata were very distant from the primary target glomerulus. Second, the primary target glomerulus was innervated only sparsely, such that the second recorded cell was either unlikely to project to the same glomerulus, or if it did, targeted different glomerular subdomains and showed no overlap. To perform patching experiments of distant cells from these two clusters successfully, previous labeling and visualization of cells, for example, as suggested by Schwarz et al. (2018) could be helpful in future studies.

### Cluster B and G drive local juxtaglomerular INs

**Figure 5.**
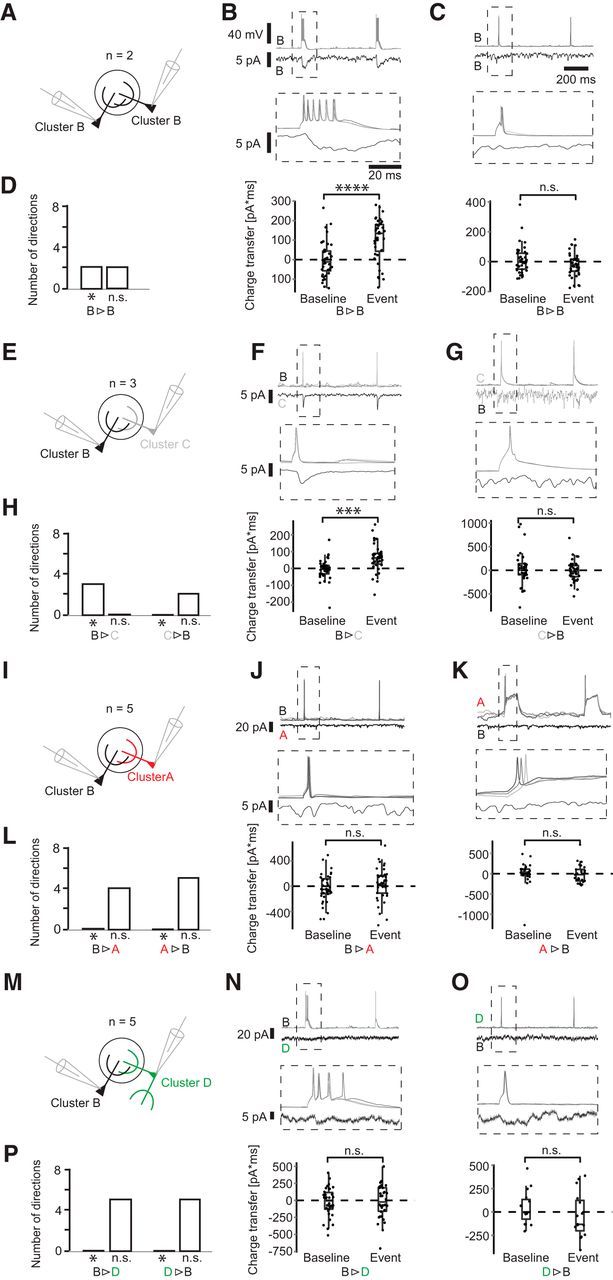
Synaptic connections of Cluster B neurons. ***A***, Schematic of simultaneous recordings of the configuration B-B. ***B***, ***C***, Short, high-amplitude current injections elicited APs in the first cell, and the corresponding current was monitored in voltage clamp. Here, current averages are shown. Middle column represents blowup of the ROI. Bottom column represents charge transfer during a 50 ms interval after spike onset compared with a randomly drawn baseline. ***D***, Histogram represents the number of significant and nonsignificant responses in each pair configuration in both directions. Asterisk indicates significance *p ≤ 0.05; ***p ≤ 0.001; ****p ≤ 0.0001; paired *t* test, for exact values, see https://figshare.com/s/7da895122de6ea83e655). ***E–P***, Paired recordings of configurations B-C, B-A, and B-D.

**Figure 6.**
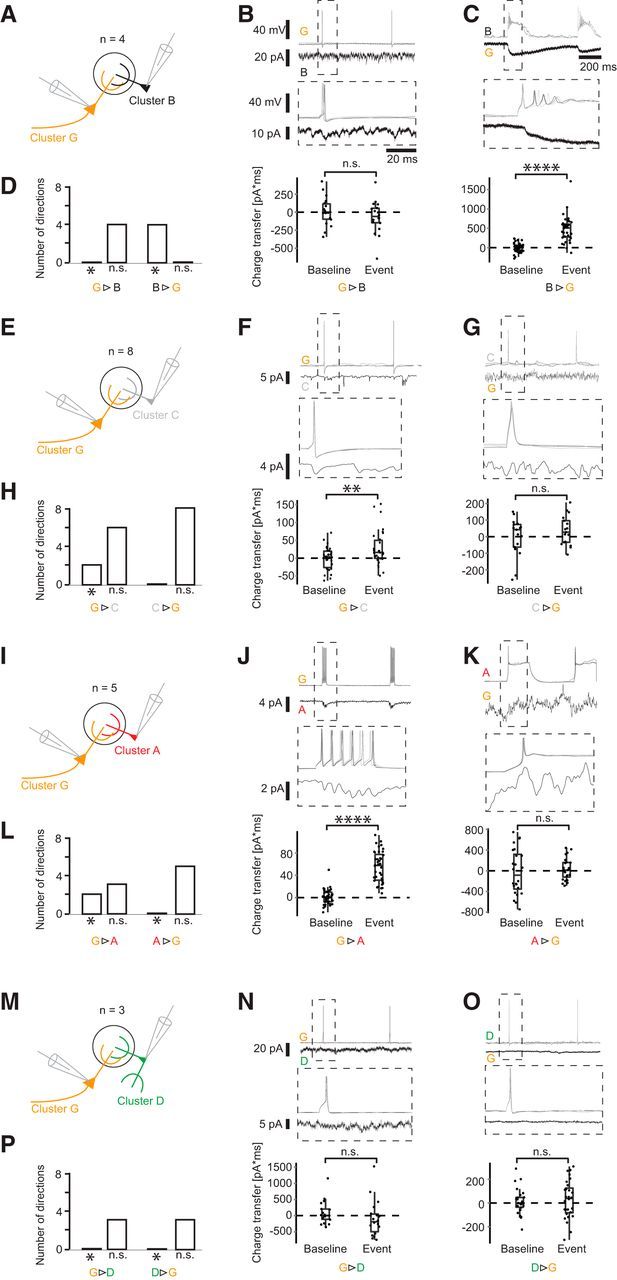
Synaptic connections of Cluster G neurons. ***A***, Schematic of paired recordings of the configuration G-B. ***B***, ***C***, Short, high-amplitude current injections elicited APs in the first cell, and the corresponding current was monitored in voltage clamp. Here, current averages are shown. Middle column represents blowup of the ROI. Bottom column represents charge transfer during a 50 ms interval after spike onset compared with a randomly drawn baseline. ***D***, Histogram showing the number of significant and nonsignificant responses in each pair configuration in both directions. Asterisk indicates significance (*p ≤ 0.05; **p ≤ 0.001; ****p ≤ 0.0001); (paired *t* test, for exact values; https://figshare.com/s/7da895122de6ea83e655). ***E–P***, Paired recordings of configurations G-C, G-A, and G-D.

Current models of glomerular circuitry found ETCs at the center of excitatory glomerular activity, driving mitral cells (MCs), JGCs, and other ETCs (Hayar et al., 2004a,b, 2005; Murphy et al., 2005; De Saint Jan et al., 2009; Najac et al., 2011; Gire et al., 2012). A further subtype, the VGLUT3^+^ ETC, was found to excite TCs, but not MCs (Tatti et al., 2014). We thus evaluated the connectivity of the 8 different pair configurations to dissect the microcircuits established by the identified cells of the defined clusters. To investigate whether a postsynaptic cell exhibited a significant current response, we compared the baseline corrected current integral in a 50 ms time interval after spike onset in each trial with a random current integral of the same length with a paired *t* test (for details, see Materials and Methods). We found that 37% (13 of 35) of neuron pairs projecting dendrites to a common target glomerulus showed a positive current transfer in the postsynaptic cell following an AP in the presynaptic cell (average latency = 1.95 ± 0.44 ms), implying an excitatory synaptic connection between the cells. Although most pairs (33 of 35) were tested in both directions, all excitatory connections found were unidirectional. Of the 8 pair configurations examined, 5 configurations formed excitatory synapses (B>G, B>C, B>B, G>C, G>A), whereas 3 configurations showed no significant responses in the postsynaptic neurons in either direction (G/D, B/D, B/A). All excitatory connections were found when either Cluster B (9 of 19 pairs with the involvement of Cluster B; Fig. 5) or Cluster G (4 of 16 pairs with the involvement of Cluster G; Fig. 6) cells were the presynaptic element within a pair.

**Figure 7.**
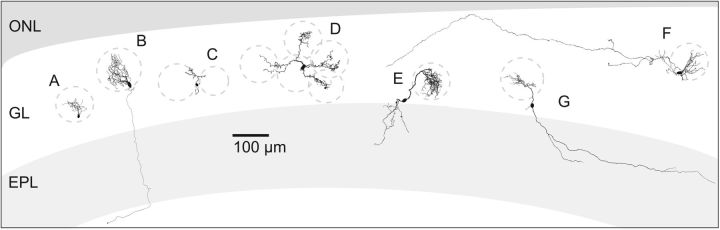
The position of example cells from each cluster in the GL. Cluster A, red cluster, microglomerular, MG cell. Cluster B, black cluster, external tufted, ET cell. Cluster C, gray cluster, uniglomerular, UG cell. Cluster D, green cluster, oligoglomerular, OG cell. Cluster E, yellow cluster, vertical superficial tufted, vST cell. Cluster F, blue cluster, polyglomerular, POG cell. Cluster G, orange cluster, horizontal superficial tufted, hST cell.

**Figure 8.**
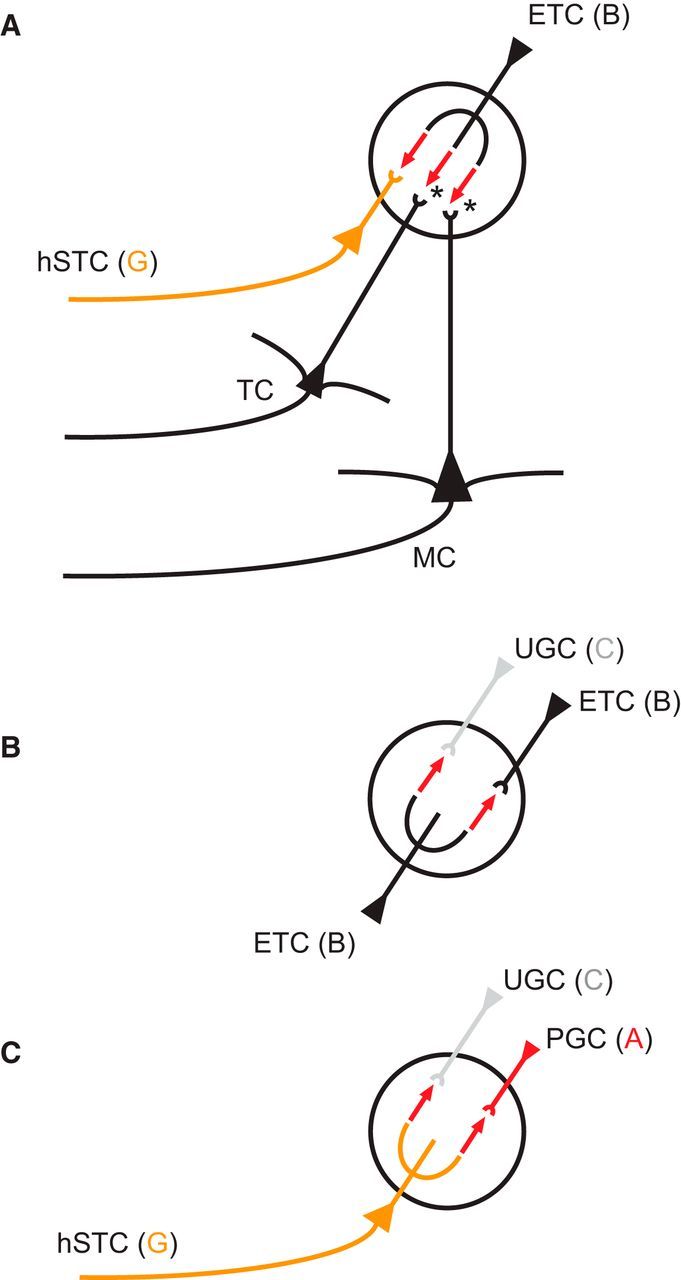
Schematic of excitatory microcircuits in the GL. ***A***, Three glomerular output pathways are driven by ETCs. We have shown ETCs forming direct excitatory synapses onto lateral dendrite-bearing STCs, hSTCs. Asterisks indicate the other two output pathways mediated by ETCs found by previous studies: MCs are driven by the OSN-ETC-MC circuit (Hayar et al., 2004a,b, 2005; Murphy et al., 2005; De Saint Jan et al., 2009; Najac et al., 2011; Fukunaga et al., 2012; Gire et al., 2012), and TCs are excited by VGLUT3^+^ ETCs (Tatti et al., 2014). ***B***, Two intraglomerular circuits are mediated by ETCs. In agreement with other studies, we find that ETCs form excitatory connections onto other ETCs (Hayar et al., 2005). Furthermore, we have shown direct excitatory synapses of ETCs onto UGCs. ***C***, Two intraglomerular circuits are mediated by lateral dendrite-bearing STCs, hSTCs. We found excitatory microcircuits of hSTCs onto MCs and UGCs, indicating that, next to ETCs, hSTCs are a second excitatory element of the glomerulus.

In all experiments (4 of 4) of the pair configuration Cluster B/G (Fig. 6*A–D*), APs elicited in the Cluster B cell resulted in excitatory responses in the Cluster G cell. APs in the Cluster G cell, however, evoked no response in Cluster B cells. Of the configuration B/B, in 2 of 2 experiments, a postsynaptic current response was found (Fig. 5*A–D*). Even though in this pair configuration both cells originated from the same cell cluster, a unidirectional connection was found in both experiments. For the pair configuration B/C, the Cluster C cell showed an excitatory response in all 3 of 3 experiments (Fig. 5*E–H*). At the same time, Cluster C also exhibited a postsynaptic current response to Cluster G as a presynaptic neuron in 2 of 8 cases (Fig. 6*E–H*). Furthermore, Cluster G formed excitatory synapses onto Cluster A cells in 2 of 5 experiments (Fig. 6*I–L*), whereas Cluster B did not evoke any response in Cluster A cells (5 of 5; Fig. 5*I–L*). The pair configurations involving Cluster D (B/D and G/D) showed no synaptic response for either Cluster B or G and no response when Cluster D was the presynaptic neuron (Figs. 5*M–P*, 6*M–P*). In summary, these results indicate that the cells of the two Clusters B and G (Fig. 7) are the main excitatory neurons for driving local INs of Cluster A and C, whereas the locally more extensive Cluster D did not receive any inputs from Cluster B and G (Fig. 8). Furthermore, the local, but densely ramifying, cells of Cluster B were the main source of local input to the extensive PNs with a lateral dendrite of Cluster G.

## Discussion

### Four types of juxtaglomerular INs within the OB GL

Broad functional and morphological variability of neurons is a key characteristic of vertebrate brain networks (Parra et al., 1998; Markram et al., 2004; Marder, 2011; Kepecs and Fishell, 2014), also present in the GL, the most superficial layer of the OB. Here, we drew a broad sample of 95 JGCs containing both PNs and INs and measured detailed morphological and physiological properties. We identified seven morphological clusters after submitting our data to hierarchical CA. The Clusters A, C, D, and F were consistent with previous descriptions of INs: Cluster A and C, the smallest clusters of the set, were in agreement with the current definition of PGCs, and because of their local confinement, to uniglomerular cells (UGCs) (Kiyokage et al., 2010). Notably, Clusters A and C distinctly differed from each other in physiological and morphological properties, suggesting that the class PGC indeed consists of two morphologically and physiologically distinct populations. While Cluster C cells were more extensive than those of Cluster A, their physiology was consistent with that of PGCs with fast regular spiking patterns (McQuiston and Katz, 2001; Ascoli et al., 2008; Najac et al., 2015). On the contrary, Cluster A cells exhibited unique plateau spiking. A qualitatively similar activity pattern has been described in PGCs of the rat OB (Puopolo and Belluzzi, 1998; Masurkar and Chen, 2011), however, requiring 20-fold larger currents for spike responses as Cluster A cells in mice. Cells exhibiting these plateau potentials have not been morphologically characterized or described as a distinct JGC class. We suggest the terms “microglomerular” cell for Cluster A and “uniglomerular” cell (Kiyokage et al., 2010) for Cluster C. Neurons of Cluster D were more extensive than those of Cluster A and C but locally confined to 2–6 glomeruli, most consistent with the concept of an oligoglomerular cell (OGC) (Kiyokage et al., 2010). Finally, the cells of Cluster F comprised the most extensive JGCs, contacting a large number of glomeruli across large distances, consistent with superficial short axon cells (Aungst et al., 2003), which are also called polyglomerular cells (POGC) (Kiyokage et al., 2010).

While our sample includes a wide and random sample of cells, technical limitations of the patch-clamp technique can distort the numbers of cells in different classes. Successful patch-clamp recordings with good access are more difficult to achieve in small cells, and recovery rates after recording are worse, possibly due to a more limited reserve of cellular resources, which are needed for survival. The relative number of cells in different classes may not be representative due to the potential undersampling of small cells. However, our sample includes cells with diameters ranging from the smallest PG cells (<6 μm) to large neurons (>15 μm); therefore, it is likely that, apart from potential very rare types, our sample includes all cell varieties.

### Relationship of the four JGC clusters to molecular markers

The four above-presented JGC clusters share common characteristics with previously described morphological cell types, expressing immunohistochemical markers, such as calretinin (CR), calbindin (CB), and tyrosine hydroxylase (TH), which have been reported to be mutually exclusive (Kosaka and Kosaka, 2007; Sawada et al., 2011). The smallest cell type in the GL are CR^+^ cells (Kosaka and Kosaka, 2007; Parrish-Aungst et al., 2007; Whitman and Greer, 2007; Benito et al., 2018), which in terms of soma size and morphological appearance correspond to the homogeneous class of cells defined by Cluster A (Table 5). A recent preprint study by Benito et al. (2018) furthermore relates CR^+^ cells directly to a homogeneous population of cells with distinct plateau potential APs, which show great agreement with Cluster A cells. According to previous studies, the second smallest cell type in the GL are CB^+^ cells (Kosaka and Kosaka, 2007; Parrish-Aungst et al., 2007; Whitman and Greer, 2007), which would match Cluster C or uniglomerular cells with comparable sizes within our data, such that Cluster C cells likely represent CB^+^ neurons. Thus, the two classes CB^+^ and CR^+^ cells are not only mutually exclusive regarding their molecular markers, but our data show that likely they are exclusive in regard to their morphological and physiological features. Both CB^+^ and CR^+^ cells have been described to avoid the olfactory nerve (ON) compartment and were thus considered Type II PG cells (Kosaka et al., 1997; Shao et al., 2009). Thus, it is likely that Cluster A (MGCs) and Cluster C (UGCs) together represent the Type II category, not receiving direct inputs from ONs.

**Table 5.** Summary of the clusters in relation to the literature

	Cluster A	Cluster B	Cluster C	Cluster D	Cluster E	Cluster F	Cluster G
Proposed types (current study)	Microglomerular	ET	Uniglomerular	Oligoglomerular	Vertical superficial tufted	Polyglomerular	Horizontal superficial tufted
Corresponding previous nomenclature	Periglomerular	ET (Pinching and Powell, 1971)	Periglomerular (Pinching and Powell, 1971)	Periglomerular	Superficial tufted or ET with basal dendrite (Antal et al., 2006)	Superficial short axon (Pinching and Powell, 1971; Aungst et al., 2003)	Superficial tufted or ET with basal dendrite (Wachowiak and Shipley, 2006; Antal et al., 2006)
			Uniglomerular (Kiyokage et al., 2010)	Oligoglomerular (Kiyokage et al., 2010)		Polyglomerular (Kiyokage et al., 2010)	
Extension	Tuft within parts of 1 glomerulus	Extensive tuft within 1 glomerulus	1–3 glomeruli	2–6 glomeruli	Tuft within 1 glomerulus, basal dendrites extending in EPL below the soma	≥7 glomeruli	Tuft within 1 glomerulus, lateral dendrite extending parallel to the GL
Median soma area (μm^2^)	42.2	90.0	55.9	87.5	98.9	79.0	107.9
Median some diameter (μm)	6.4	9.4	7.4	9.3	9.8	8.8	10.3
Input (current study)	hSTC	ETC	ETC, hSTC				ETC
Input (from the literature)	Unknown	OSN, ETC	ETC, TC, MC	ETC, OSN	Unknown	ETC, OSN	Unknown
Function	Unclear	Feedforward excitation (Hayar et al., 2004a, 2005; De Saint Jan et al., 2009; Gire et al., 2012; Tatti 2014)	Feedback and feedforward inhibition (Murphy et al., 2005; Shao et al., 2009; Gire et al., 2012; Najac et al., 2015)	Lateral inhibition between local glomeruli (Kiyokage et al., 2010; Whitesell et al., 2013)	Unknown	Lateral inhibition between distant glomeruli (Aungst et al., 2003; Whitesell et al., 2013)	Feedforward excitation (current study)
Expected molecular subtypes	CR, GAD65 (Kosaka et al., 2007; Parrish-Aungst et al., 2007; Whitman and Greer, 2007; Kiyokage et al., 2010)	VGLUT2, VGLUT3 (Tatti et al., 2014)	CB, GAD65 (Kosaka et al., 2007; Parrish-Aungst et al., 2007; Whitman and Greer, 2007)	TH, GAD67 (Kosaka et al., 2007; Parrish-Aungst et al., 2007; Whitman and Greer, 2007; Kiyokage et al., 2010)	Unknown	TH, GAD67 (Kosaka et al., 2007; Parrish-Aungst et al., 2007; Whitman and Greer, 2007; Kiyokage et al., 2010)	Vasopressin (Tobin et al., 2010)

Of the three mutually exclusive marker types, TH^+^ were reported to have the largest somatic size (Kosaka and Kosaka, 2007; Parrish-Aungst et al., 2007; Whitman and Greer, 2007), with dendrites extending within and outside the ON compartment of the glomerulus, thus classified as Type I PG cells (Kosaka et al., 1997). This description fits our data of Cluster D and F neurons of OGCs and POGCs, which also had the largest somatic sizes of glomerular INs within our data, likely representing the TH^+^ population. These neurons receive direct ON inputs and ET inputs and project dendrites across several glomeruli, playing a key role in inhibitory interglomerular coordination (Kiyokage et al., 2010; Whitesell et al., 2013). A similar role was attributed to superficial short axon cells or polyglomerular cells, which project the longest distances across glomeruli and best compare with the cells defined by Cluster F (Aungst et al., 2003; Kiyokage et al., 2010; Whitesell et al., 2013).

In regard to neurotransmitter enzyme expression, glutamic acid decarboxylase (GAD) 65 and 67 have been used to classify cells, however, with some overlap between the expressed markers across chemotypes. Because the majority of small, uniglomerular cells express GAD65^+^ (Kiyokage et al., 2010), it is likely that Clusters A and C fall into that same category. Similarly, it is plausible that neurons that were described as TH^+^/GAD67^+^ correlate with the cells from Cluster D and F.

### Three types of PNs within the superficial EPL and deep GL

TCs located within the superficial EPL or the deep GL are subdivided into two classes by morphological criteria: the ETC without basal dendrites (Pinching and Powell, 1971; Macrides and Schneider, 1982; Hayar et al., 2004a; De Saint Jan et al., 2009; Tatti et al., 2014) and the ETC with basal dendrites, which has also been referred to as the superficial tufted cell (STC) (Macrides and Schneider, 1982; Nagayama et al., 2014). The basal dendrite-bearing cells were qualitatively separated into two types: one with a lateral dendrite and the other with locally branching dendrites beneath the cell (Antal et al., 2006). We found the morphologies of Clusters B, E, and G to be consistent with these current descriptions of PNs. The attributes of Cluster B neurons closely match descriptions of classic ETCs without basal dendrites. They were confined to one glomerulus in which they extensively branched and exhibited bursting spiking behavior (Macrides and Schneider, 1982; Hayar et al., 2004a; De Saint Jan et al., 2009; Masurkar and Chen, 2012). The PNs of Clusters E and G best correlate with definitions of basal dendrite-bearing ETCs. The classic STC is represented by Cluster G, bearing a lateral dendrite, while Cluster E cells projecting locally beneath the soma best correspond to those described by Antal et al., 2006. These, however, have not been described as a quantitatively separate cluster and have not yet received a name. We suggest a differentiation of dendrite-bearing ETCs into horizontal STCs and vertical STCs, and in agreement with previous studies (Hayar et al., 2004a; Tobin et al., 2010; Nagayama et al., 2014) would reserve the term ETC to the classic type not bearing any basal dendrites.

### Objective prediction of cell identities based on population-wise data

Similarly to the morphological classification, CA of physiological parameters revealed seven discrete cell classes. While MGC and ETC morphotypes and physiotypes (A/I and B/II) were perfectly predictive of each other, UGCs and OGCs showed notable variability regarding physiological properties, in accordance with the view that in certain IN populations functional properties are distributed in a continuum (Marder, 2011; Otopalik et al., 2017), which also seems to hold true for UGCs and OGCs. Furthermore, additional subclasses may exist within our dataset, for which the measured morphological parameters and the defined clusters were not sensitive, a limitation inherent to the method of cluster analysis and gap statistics.

Achieving a high population-wise predictability of cell identity is nonetheless crucial for intrastudy and interstudy comparability. Especially in typically long-duration paired-recording experiments, the complete morphological reconstruction of neurons is often not sufficiently reproducible, and relying on detailed morphological parameters to identify cell classes is not possible. We therefore identified a set of parameters, including five physiological parameters and one morphological parameter, which could reliably predict the cell clusters in recorded cell pairs, despite the physiological variability of some classes. The physiological parameters were chosen to be reliably attainable during well-controlled *in vivo* experiments as previously shown (Fukunaga et al., 2012; Kollo et al., 2014; Jordan et al., 2017). We have further included parameters that are less sensitive to series resistance changes occurring during long-duration and *in vivo* recordings, such as the AHP shape (Kollo et al., 2014) and the AP speed ratio. With an objective method for the identification of the neurons in each pair, we could investigate the functional connectivity between clusters.

### Novel pathway of ETC-driven glomerular output through horizontal STCs

We identified the cluster identity of each pair of neurons in whole-cell patch-clamp experiments by using the model mentioned above based on an SVM classifier. Consistent with previous studies, we found that ETCs are at the heart of excitatory glomerular activity (Hayar et al., 2004a,b, 2005; Murphy et al., 2005; De Saint Jan et al., 2009; Najac et al., 2011; Gire et al., 2012), driving PNs and UGCs. However, we found no evidence for excitatory input from ETCs to two other populations of JGCs, OGCs, and MGCs. Nagayama et al. (2014) emphasized in their detailed review on the topic the importance of studying functional differences between classic ETCs without lateral dendrite and STCs with a lateral dendrite. Here, we show that the two cell types are not only distinct in morphotype, physiotype, and connectivity but even find that they are functionally connected with each other. Indeed, ETCs do not only drive UGCs and other classic ETCs, but also form excitatory synapses onto these horizontal superficial tufted cells (hSTCs). Thus, our results indicate that, next to MCs (ET-driven) and TCs (VGLUT3^+^ ET-driven), hSTCs represent a third class of ET-driven PNs (Fig. 8). The targeted local INs provide additional evidence supporting functional differences between ETCs and hSTCs. While we show that both clusters directly excite UGCs and neither forms synapses with OGCs, ETCs additionally drive other ETCs but do not drive MGCs (Fig. 8). On the contrary, hSTCs provide input to MGCs and do not excite ETCs. Consequently, hSTCs, next to ETCs (Hayar et al., 2004a), represent a second excitatory element of local JGC recruitment.

### Novel pathway of juxtaglomerular IN excitation through hSTCs

MGCs, UGCs, and OGCs differ in the synaptic inputs they receive from ETCs and hSTCs, supporting the assertion that they form three functionally relevant groups of JGCs. While we found MGCs to receive excitatory inputs from hSTCs, we found no evidence for inputs from ETCs (Fig. 8). UGCs, however, were driven by both ETCs and hSTCs. In contrast to UGCs and MGCs, OGCs were neither ET- nor hST-driven. These findings are in agreement with existing data about a differentiation into ET-driven PGCs and ON-driven PGCs (Shao et al., 2009). Whereas UGCs are ET-driven and hST-driven, MGCs are hST-driven only, and OGCs are neither ET- nor hST-driven. Thus, next to ON-driven and ET-driven PGCs, MGCs may potentially represent a third functionally different type, the hST-driven cell. Notably, in all of the bidirectional paired experiments, no inhibitory currents could be measured, even though in 29 of 35 of the experiments one of the cells was a local neuron from Clusters A, C, or D, thus putatively representing inhibitory INs. Because we chose to investigate bidirectional synapses of cell pairs, the internal solutions of the putative postsynaptic neurons were not changed to high-chloride internal solutions, which could have unmasked inhibitory currents that are evoked by small INs within the larger, low-resistance neurons from Cluster B and G.

### Correlative classification of physiotypes and morphotypes

With recent advancements in *in vivo* electrophysiology (Kodandaramaiah et al., 2012; Annecchino et al., 2017) and volume electron microscopy (Briggman and Denk, 2006; Briggman and Bock, 2012; Denk et al., 2012; Helmstaedter, 2013), the understanding of neuronal microcircuits has become more obtainable. However, neither of these techniques alone can provide sufficient information to build effective models of circuit function. While experiments with correlated physiology and connectomics provide the most information, numerous uncorrelated datasets exist. Here we provide a population-wise database, which given a defined set of morphological parameters from volume reconstructions, can be reliably used to predict a morphological class and provide a range of its corresponding physiological parameters. We further demonstrate that a combination of physiological and morphological factors obtainable in *in vitro* and *in vivo* experiments can be used to reliably predict the morphological class of neurons in the GL.
